# A Simplified Metric to Streamline Between-Group Fairness Assessment for Predictive Models: Algorithm Development and Evaluation Study

**DOI:** 10.2196/85822

**Published:** 2026-08-24

**Authors:** Haoyuan Wang, Chuan Hong, Michael Pencina, Matthew Engelhard

**Affiliations:** 1 Department of Biostatistics and Bioinformatics, Duke University School of Medicine, 2424 Erwin Rd, Durham, NC, 27705, United States, 1 919-613-3665

**Keywords:** trustworthy AI, performance metrics, cardiovascular risk prediction, machine learning, algorithms

## Abstract

**Background:**

Fairness evaluation is essential for trustworthy clinical risk prediction. However, existing fairness-oriented discrimination metrics either ignore cross-group comparisons or rely on exhaustive pairwise evaluations, making them difficult to interpret and impractical for model selection.

**Objective:**

This study aimed to develop and evaluate novel fairness-oriented discrimination metrics for clinical risk prediction that address limitations of within-group and pairwise cross-group approaches.

**Methods:**

We examined theoretical properties of existing U-statistic–based metrics, including concordance index (CI) and area under the receiver operating characteristic curve (AUC), when applied to subgroups. We highlighted the distinction between within-group discrimination (ranking within a subgroup) and group-level discrimination (ranking relative to the broader population). Building on this framework, we proposed group-level extensions of the CI and AUC that summarize subgroup-specific performance in a single interpretable measure. We then applied these metrics to the PREVENT (Predicting Risk of Cardiovascular Disease Events) equation, a recently developed model for atherosclerotic cardiovascular disease.

**Results:**

The traditional subgroup-specific CI and AUC captured within-group but not group-level discrimination, obscuring inequities in clinical decision-making. Existing cross-group approaches (eg, the xCI and xAUC metrics) addressed this limitation but became computationally and interpretively burdensome with multiple subgroups due to pairwise comparisons. Our proposed metrics provided a streamlined alternative, yielding 1 summary statistic per subgroup while retaining sensitivity to cross-group ranking disparities. Applied to PREVENT, these metrics revealed differences in subgroup performance not apparent from within-group evaluations.

**Conclusions:**

By distinguishing between within-group and group-level discrimination, our framework clarifies a common source of misinterpretation in fairness evaluation. The proposed group-level extensions of the CI and AUC provide practical, interpretable tools for evaluating fairness in clinical prediction models, enabling more transparent and equitable risk assessment.

## Introduction

Clinical risk prediction models are typically deployed as continuous risk scores that rank patients by their likelihood of future events and support downstream actions such as preventive therapy, screening, and care management. Because these tools are often used for prioritization, discrimination is commonly evaluated using U-statistic–based metrics of order 2, including the Harrell concordance index (CI) for time-to-event outcomes and the area under the receiver operating characteristic curve (AUC) for binary outcomes [1-3]. These metrics quantify the probability that the model will correctly order a randomly selected comparable pair, providing a direct measure of ranking quality that aligns with how risk scores are used in many clinical workflows.

In real-world health care deployments, strong overall discrimination does not guarantee equitable performance across patient subgroups. Models can create or amplify inequities when their errors, calibration, or ranking behavior differ across groups defined by race, sex, and other sociodemographic characteristics. This concern is central to the machine learning literature on fairness in prediction and algorithmic accountability, which emphasizes that fairness is multidimensional and that different fairness definitions reflect different normative goals [4-6]. In the literature, a standard approach is model auditing: evaluating a deployed model using a set of complementary fairness metrics such as demographic parity [7], equalized odds [7], and parity in calibration [8,9].

While the literature offers robust fairness definitions and frameworks, the methodology for applying the CI and AUC to fairness evaluation is far less developed. A common approach in subgroup fairness evaluation is to compute the CI or AUC separately within each subgroup. While straightforward and widely used, this method evaluates the model’s ability to correctly rank individuals in a given subgroup relative to others in the same subgroup (referred to as within-group discrimination) rather than its ability to correctly rank them relative to all individuals across the broader population, including those in other subgroups (referred to as group-level discrimination).

This distinction is critical: within-group metrics measure how individuals rank relative only to others in the same group, ignoring how they compare to those outside their group. In real-world settings such as clinical decision-making, the same decision rules, such as thresholds for initiating treatment or screening, are typically applied across groups. As a result, what counts as high or low risk is defined relative to the broader population, not just within a group. Therefore, within-group discrimination may be a woefully incomplete measure of fairness as it fails to reveal whether a model systematically treats a group differently from the broader population, even if it accurately distinguishes risk within the group.

However, much of the existing literature has either overlooked this distinction [10,11] or misinterpreted within-group metrics as indicators of group-level performance [12], leading to misleading conclusions about fairness. Recent work, such as on the cross-group AUC (xAUC) [13] and cross-group CI (xCI) [14] metrics, has recognized that looking at ranking performance across groups is important. These metrics improve upon within-group approaches by conditioning on group membership to evaluate how well a model ranks individuals between different groups. However, because they rely on pairwise comparisons across all subgroups, they become unwieldy when multiple groups are present, resulting in *k*^2^ total comparisons for *k* subgroups. This complexity limits their interpretability and scalability. Therefore, there is a need for an intermediate approach: one that retains the ability to assess cross-group ranking performance yet simplifies interpretation by providing a single fairness measure per subgroup (ie, *k* total) rather than an exhaustive set of pairwise comparisons.

In this work, we propose novel group-level extensions of commonly used discrimination metrics, which address key limitations of within-group and pairwise cross-group approaches by providing a single, interpretable summary of group-specific performance. We then demonstrate the utility of our proposed metrics by applying them to evaluate the group-specific performance of the PREVENT (Predicting Risk of Cardiovascular Disease Events) equation [15], a recently developed risk prediction model for atherosclerotic cardiovascular disease (ASCVD).

## Methods

In this section, we revisit traditional discrimination metrics (CI and AUC), formally define their within-group and cross-group counterparts, and introduce our proposed group-level extensions (gCI for cCI and gAUC for AUC) that directly capture a model’s discriminative performance for specific subgroups relative to the population.

### Overall Discrimination

Both the CI and AUC can be interpreted as U-statistics of order 2, estimating the probability that the model will assign higher predicted risk to an individual who experiences the event earlier (CI) or to a case over a control (AUC).

The CI is defined as follows: CI = *P*(*R*_*i*_ > *R*_*j*_ | *T*_*i*_ < *T*_*j*_), where *R*_*i*_ and R_*j*_ are the predicted risk scores and *T*_*i*_ and *T*_*j*_ are the true event times for individuals *i* and *j*.

In the presence of censoring, we estimate the CI by restricting to comparable pairs. If *o*_*i*_ denotes the observed time (event or censoring) and *y*_*i*_ ∈ {0, 1} denotes the event indicator (*y*_*i*_=1 if the event was observed), for the CI, comparable pairs are defined as *S*_CI_ = {(*i*, *j*) : *y*_*i*_=1, *o*_*i*_ < *o*_*j*_}, and concordant pairs are defined as *S*^*c*^_CI_ = {(*i*, *j*) ∈ *S*_CI_ : *R*_*i*_ > *R*_*j*_}. The empirical CI is ĈI = |*S*^*c*^_CI_| / |*S*_CI_|.

The AUC is defined as AUC = *P*(*R*_*i*_ > *R*_*j*_ | *Y*_*i*_ = 1, *Y*_*j*_ = 0), where *R*_*i*_ and *R*_*j*_ are predicted risk scores and *Y*_*i*_, *Y*_*j*_ ∈ {0, 1} are binary outcome labels (1=event; 0=nonevent). For this metric, comparable pairs are defined as *S*_AUC_ = {(*i*, *j*) : *Y*_*i*_ = 1, *Y*_*j*_ = 0}, and concordant pairs are defined as *S*^*c*^_AUC_ = {(*i*, *j*) ∈ *S*_AUC_ : *R*_*i*_ > *R*_*j*_}. The empirical AUC is ÂUC = |*S*^*c*^_AUC_| / |*S*_AUC_|.

### Within-Group Discrimination

When individuals are classified into groups *G*_*i*_ ∈ *G*, a common approach to subgroup fairness evaluation is to compute discrimination metrics separately within each group. For a given group *m*, both the CI and AUC can be defined conditionally on group membership.

The within-group CI is CI(*m*) = *P*(*R*_*i*_ > *R*_*j*_ | *T*_*i*_ < *T*_*j*_, *G*_*i*_ = *m*, *G*_*j*_ = *m*), and the within-group AUC is AUC(*m*) = *P*(*R*_*i*_ > *R*_*j*_ | *Y*_*i*_ = 1, *Y*_*j*_ = 0, *G*_*i*_ = *m*, *G*_*j*_ = *m*). These metrics are estimated by restricting to comparable pairs where both individuals belong to group *m*. Specifically, the comparable pairs for the CI and the AUC are *S*_CI_(*m*) = {(*i*, *j*) : *y*_*i*_ = 1, *o*_*i*_ < *o*_*j*_, *G*_*i*_ = *m*, *G*_*j*_ = *m*} and *S*_AUC_(*m*) = {(*i*, *j*) : *Y*_*i*_ = 1, *Y*_*j*_ = 0, *G*_*i*_ = *m*, *G*_*j*_ = *m*}, respectively, and the concordant pairs for the CI and the AUC are *S*^*c*^_CI_(*m*) = {(*i*, *j*) ∈ *S*_CI_(*m*) : *R*_*i*_ > *R*_*j*_} and *S*^*c*^_AUC_(*m*) = {(*i*, *j*) ∈ *S*_AUC_(*m*) : *R*_*i*_ > *R*_*j*_}, respectively. The empirical estimators are ĈI(*m*) = |*S*^*c*^_CI_(*m*)| / |*S*_CI_(*m*)| and ÂUC(*m*) = |*S*^*c*^_AUC_(*m*)| / |*S*_AUC_(*m*)|, respectively.

These within-group metrics assess discrimination only among individuals within the same group, ignoring cross-group comparisons that may also be critical for evaluating fairness and overall model performance.

### Cross-Group Discrimination

The xCI and xAUC metrics incorporate the notion of cross-group discrimination by conditioning on the group membership of both individuals in each comparable pair. For example, when considering 2 groups *m* and *n*, the cross-group CI is xCI(*m*, *n*) = *P*(*R*_*i*_ > *R*_*j*_ | *T*_*i*_ < *T*_*j*_, *G*_*i*_ = *m*, *G*_*j*_ = *n*), and the cross-group AUC is xAUC(*m*, *n*) = *P*(*R*_*i*_ > *R*_*j*_ | *Y*_*i*_ = 1, *Y*_*j*_ = 0, *G*_*i*_ = *m*, *G*_*j*_ = *n*).

The comparable pairs for the CI and the AUC are defined as follows: *S*_xCI_(*m*, *n*) = {(*i*, *j*) : *y*_*i*_ = 1, *o*_*i*_ < *o*_*j*_, *G*_*i*_ = *m*, *G*_*j*_ = *n*} and *S*_xAUC_(*m*, *n*) = {(*i*, *j*) : *Y*_*i*_ = 1, *Y*_*j*_ = 0, *G*_*i*_ = *m*, *G*_*j*_ = *n*}, respectively. It should be noted that xCI(*m*, *m*) and xAUC(*m*, *m*) are the same as the within-group CI and AUC of group *m*. The concordant pairs for the CI and the AUC are defined as follows: *S*^*c*^_xCI_(*m*, *n*) = {(*i*, *j*) ∈ *S*_xCI_(*m*, *n*) : *R*_*i*_ > *R*_*j*_} and *S*^*c*^_xAUC_(*m*, *n*) = {(*i*, *j*) ∈ *S*_xAUC_(*m*, *n*) : *R*_*i*_ > *R*_*j*_}, respectively. The empirical estimators for the CI and the AUC are *x̂*CI(*m*, *n*) = |*S*^*c*^_xCI_(*m*, *n*)| / |*S*_xCI_(*m*, *n*)| and *x̂*AUC(*m*, *n*) = |*S*^*c*^_xAUC_(*m*, *n*)| / |*S*_xAUC_(*m*, *n*)|, respectively.

When there are |*G*|=*k* total groups, the xCI and xAUC metrics yield *k*^2^ values, summarizing all possible within-group and cross-group discrimination comparisons.

### Group-Level Discrimination

To more comprehensively assess a model’s discriminative performance for a specific group, we propose group-level extensions of the CI and AUC. These metrics evaluate the model’s ability to correctly rank individuals when at least one member of the pair belongs to the group of interest. For a group *A* ∈ *G*, we define the group-level CI (gCI) as gCI(*m*) = *P*(*R*_*i*_ > *R*_*j*_ | *T*_*i*_ < *T*_*j*_, *G*_*i*_ = *m* or *G*_*j*_ = *m*) and the group-level AUC (gAUC) as gAUC(*m*) = *P*(*R*_*i*_ > *R*_*j*_ | *Y*_*i*_ = 1, *Y*_*j*_ = 0, *G*_*i*_ = *m* or *G*_*j*_ = *m*).

These metrics are estimated by restricting to comparable pairs that involve at least one member from group *A*. Specifically, the comparable pairs for the gCI and gAUC metrics are defined as *S*_gCI_(*m*) = {(*i*, *j*) : *y*_*i*_ = 1, *o*_*i*_ < *o*_*j*_, *G*_*i*_ = *m* or *G*_*j*_ = *m*} and *S*_gAUC_(*m*) = {(*i*, *j*) : *Y*_*i*_ = 1, *Y*_*j*_ = 0, *G*_*i*_ = *m* or *G*_*j*_ = *m*}, respectively, and the concordant pairs are defined as *S*^*c*^_gCI_(*m*) = {(*i*, *j*) ∈ *S*_gCI_(*m*) : *R*_*i*_ > *R*_*j*_} and *S*^*c*^_gAUC_(*m*) = {(*i*, *j*) ∈ *S*_gAUC_(*m*) : *R*_*i*_ > *R*_*j*_}, respectively. The empirical estimators for the gCI and gAUC metrics are ĝCI(*m*) = |*S*^*c*^_gCI_(*m*)| / |*S*_gCI_(*m*)| and ĝAUC(*m*) = |*S*^*c*^_gAUC_(*m*)| / |*S*_gAUC_(*m*)|, respectively.

Unlike within-group metrics, the group-level indexes gCI(*m*) and gAUC(*m*) include both within-group and cross-group comparisons involving individuals from group *m*. As such, they provide a more comprehensive view of how well the model discriminates for individuals in group *m* relative to the entire population.

Additional properties of gCI and gAUC, including their relationship to xCI, xAUC, their U-statistic interpretation, and the impact of group size imbalance, are provided in Multimedia Appendix 1.

### Ethical Considerations

All study procedures were approved by the Duke University Health System Institutional Review Board. The approved protocol number is Pro00115219.

### Application to Cardiovascular Risk Prediction via PREVENT

To validate the proposed metric, we curated a comprehensive electronic health record dataset from Truveta, a real-world data platform containing records from more than 120 million patients across the United States, and used it to evaluate the PREVENT equation for predicting 10-year risk of ASCVD [15]. We applied the same inclusion and exclusion criteria established by Khan et al [15]. Risk factors were identified from structured electronic health record data using *International Classification of Diseases* and Systematized Nomenclature of Medicine codes. We then implemented the published PREVENT equation coefficients to generate predicted risk for each eligible individual. Model discrimination was then assessed via CI–based metrics (CI, xCI, and gCI) when structuring ASCVD events as a time-to-event outcome with right censoring and via AUC-based metrics (AUC, xAUC, and gAUC) when structuring it as a binary outcome. The details on cohort definition, variable curation, and model implementation have been published previously [16], and comprehensive details including model coefficients are provided in Multimedia Appendix 1.

## Results

A total of 554,675 individuals were in the study cohort, including 9858 (1.8%) with incident ASCVD and 544,817 (98.2%) without ASCVD during follow-up. As shown in Table 1, in the test set, the population size was 166,088, with a mean age of 54.3 (SD 12.6) years; 55.3% (n=91,896) were female, 69.0% (n=114,565) were White individuals, 9.4% (n=15,611) were Asian individuals, 5.5% (n=9175) were Black individuals, and 16.1% (n=26,737) were classified as other races. Mean systolic blood pressure was 125.1 (SD 13.4) mm Hg, mean total cholesterol was 195.3 (SD 35.7) mg/dL, mean high-density lipoprotein cholesterol was 55.6 (SD 15.5) mg/dL, mean BMI was 28.5 (SD 5.0) kg/m^2^, and mean estimated glomerular filtration rate was 0.9% (SD 0.3%). In addition, 8.6% (14,262/166,088) had diabetes, 9.3% (15,484/166,088) were current smokers, 22.5% (37,330/166,088) were receiving antihypertensive treatment, and 16.6% (27,501/166,088) were receiving statin treatment. The mean follow-up time was 7.1 (SD 2.7) years for ASCVD, and the observed event rates in the test set were 2.8% (4679/166,088) for total cardiovascular disease and 1.8% (2934/166,088) for ASCVD.

**Table 1.** Baseline characteristics of the study population in the test set. The table summarizes the distribution of demographic, clinical, and social determinant of health variables in the test set (n=166,088).

Variable	Values
Age (y), mean (SD)	54.3 (12.6)
Sex (female), n (%)	91,896 (55.3)
Race, n (%)	
Asian	15,611 (9.4)
Black	9175 (5.5)
Others	26,737 (16.1)
White	114,565 (69.0)
Ethnicity, n (%)	
Hispanic or Latino	18,230 (11.0)
No information	16,409 (9.9)
Not Hispanic or Latino	131,449 (79.1)
Systolic blood pressure (mm Hg), mean (SD)	125.1 (13.4)
Total cholesterol (mg/dL), mean (SD)	195.3 (35.7)
High-density lipoprotein cholesterol (mg/dL), mean (SD)	55.6 (15.5)
BMI (kg/m^2^), mean (SD)	28.5 (5.0)
Diabetes, n (%)	14,262 (8.6)
Current smokers, n (%)	15,484 (9.3)
Antihypertensive treatment, n (%)	37,330 (22.5)
Statin treatment, n (%)	27,501 (16.6)
Estimated glomerular filtration rate (%), mean (SD)	0.9 (0.3)
Cardiovascular disease follow-up time (y), mean (SD)	7.1 (2.7)
Atherosclerotic cardiovascular disease follow-up time (y), mean (SD)	7.1 (2.7)
Heart failure follow-up time (y), mean (SD)	7.1 (2.7)
Total cardiovascular disease events, n (%)	4679 (2.8)
Atherosclerotic cardiovascular disease events, n (%)	2934 (1.8)
Heart failure events, n (%)	2798 (1.7)
Insurance, n (%)	
Other insurance	49,129 (29.6)
Private insurance	94,787 (57.1)
Public insurance	22,172 (13.3)

**Figure 1.**
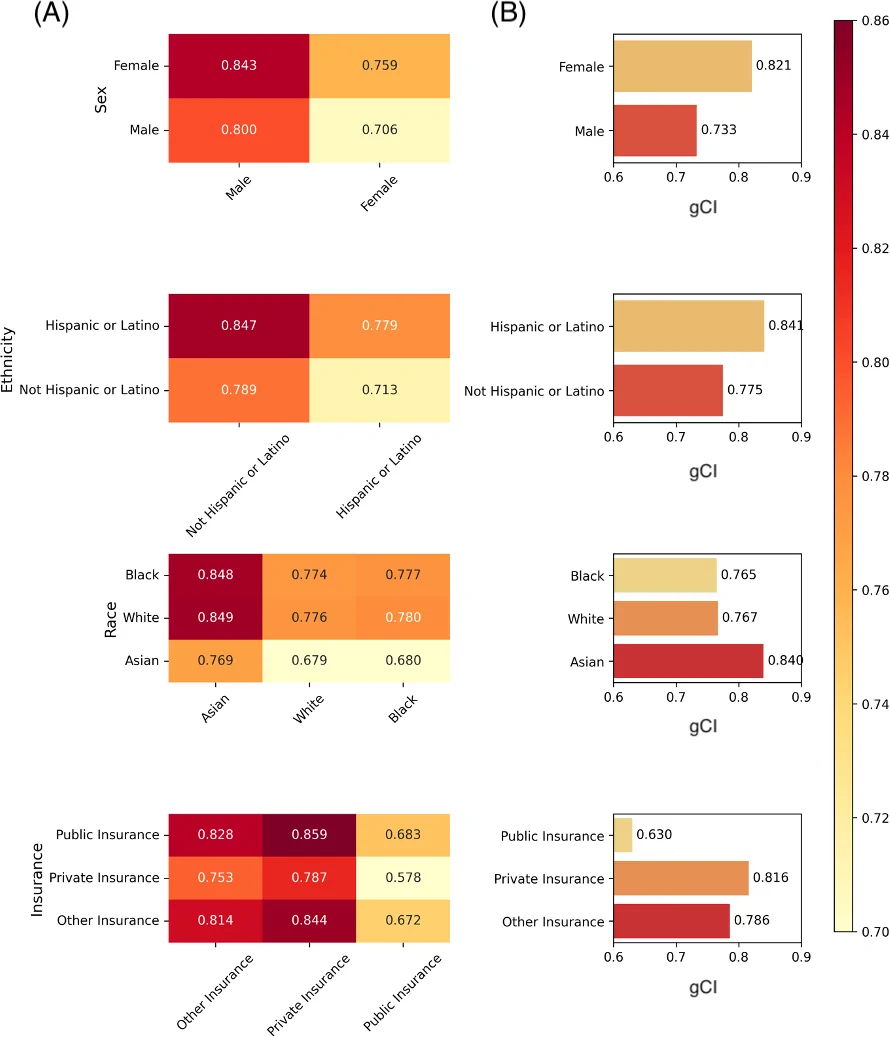
Evaluation of fairness and discrimination performance across demographic groups using the concordance index (CI). The heat maps (A) display the cross-group CI values, where each cell represents the concordance between 2 randomly selected individuals: the row corresponds to the group of the individual with a shorter observed survival time, and the column corresponds to the group of the individual with a longer survival time. Main diagonal (top left to bottom right) entries represent within-group CI values, and off-diagonal entries reflect cross-group CI values. The bar plots (B) show group-level CI values, summarizing discrimination performance for a group across all relevant comparisons. The color scale to the right indicates the magnitude of the metric values.

As shown in Figure 1, the proposed group-level metrics, gCI and gAUC, capture a different aspect of subgroup performance from that captured by the traditional within-group CI and within-group AUC. The within-group CI or AUC for a subgroup is obtained by restricting comparisons to pairs in which both individuals belong to that same subgroup; thus, it reflects how well the model ranks risk within the subgroup only. In contrast, the gCI and gAUC summarize discrimination across all comparable pairs involving that subgroup, including both within-group and cross-group comparisons. Accordingly, the gCI and gAUC quantify how well the model discriminates for members of a subgroup relative to the broader population rather than only relative to others in the same subgroup. This distinction is important for fairness assessment because clinical prediction models are typically used in shared decision environments, where patients from different groups are evaluated under the same risk framework.

For the CI-based analysis, gCI revealed disparities that were not apparent from the within-group CI alone. For example, among individuals with public insurance, the within-group CI was 0.683, whereas the gCI was lower at 0.630, indicating that the model ranked risk less well for public insurance patients when comparisons with other insurance groups were also taken into account. In contrast, private insurance had a within-group CI of 0.787 and a gCI of 0.816. As a result, the public vs private gap increased from 0.104 using the traditional within-group CI to 0.186 using the gCI, showing that cross-group comparisons exposed a substantially larger disparity in discrimination. Similar patterns were observed in other domains. For ethnicity, the within-group CI differed little between Hispanic or Latino and non–Hispanic or Latino individuals (0.779 vs 0.789; difference=0.010), yet the corresponding gCI values were 0.841 and 0.775 (difference=0.066). For race, within-group CI values were nearly identical across Black, White, and Asian participants (0.777, 0.776, and 0.769, respectively), whereas the gCI showed a wider spread (0.765, 0.767, and 0.840, respectively). These findings illustrate that a model can appear to perform similarly across groups when assessed only within a group while still exhibiting meaningful cross-group disparities once subgroup members are evaluated relative to the full population.

**Figure 2.**
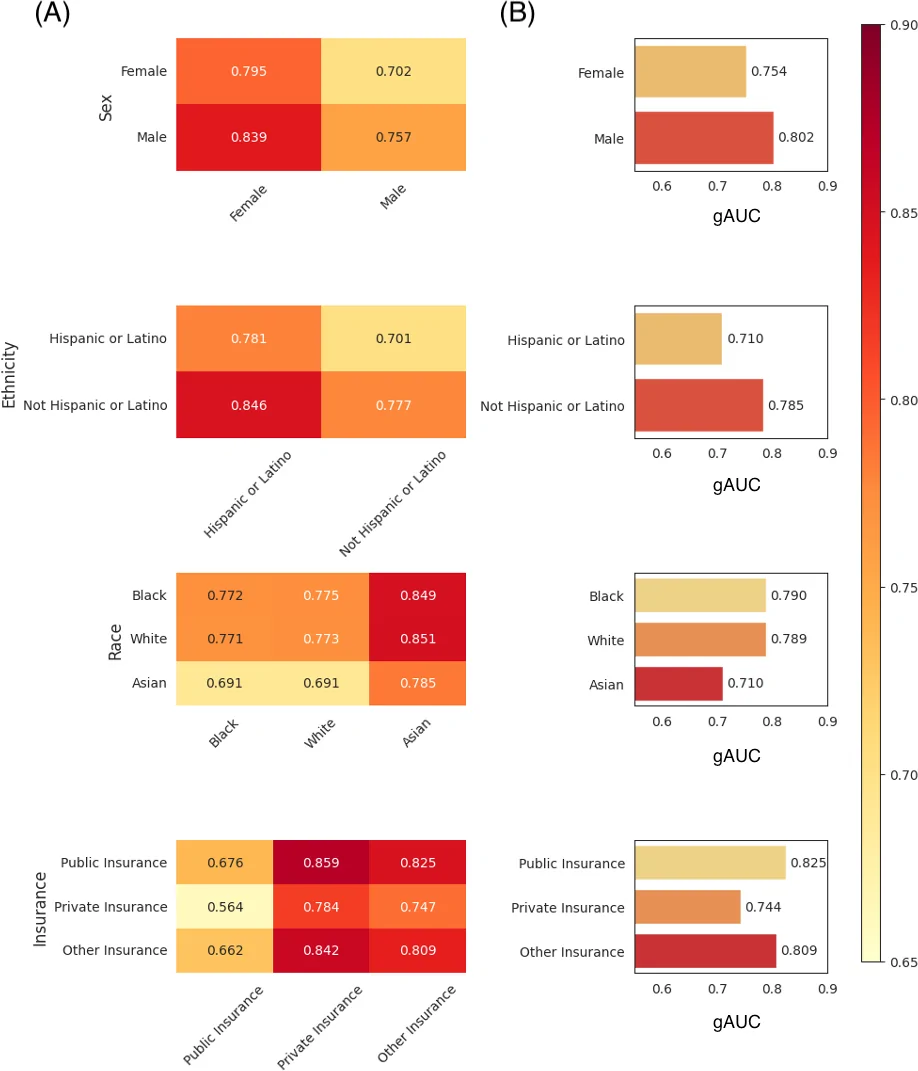
Evaluation of fairness and discrimination performance across demographic groups using the area under the receiver operating characteristic curve (AUC). The heat maps (A) display cross-group AUC values, where rows correspond to the case group and columns correspond to the control group. The main diagonal (top left to bottom right) entries represent within-group AUC values, and off-diagonal entries reflect cross-group AUC values. The bar plots (B) show group-level AUC values, summarizing discrimination performance for a group across all relevant comparisons. The color scale indicates the magnitude of the metric values.

A related but not identical pattern was observed for the binary outcome analysis using the gAUC (Figure 2). These results also suggest that the gAUC provides additional insight into ranking performance beyond what is captured by the within-group AUC alone. The most striking example was public insurance: the within-group AUC was 0.676, but the gAUC was substantially higher at 0.825. This suggests that, although discrimination among public insurance individuals alone was modest, the model more effectively distinguished case-control pairs involving public insurance individuals. For ethnicity, the within-group AUC values were almost identical for Hispanic or Latino and non–Hispanic or Latino individuals (0.781 vs 0.777; difference=0.004), whereas the gAUC values diverged much more clearly (0.710 vs 0.785; difference=0.075). For race, within-group AUC values were also fairly similar across Black, White, and Asian participants (0.772, 0.773, and 0.785, respectively), but the gAUC separated Asian participants from Black and White participants (0.710 vs 0.790 and 0.789, respectively). Thus, even when the traditional within-group AUC suggests little heterogeneity, the gAUC can reveal clinically meaningful differences in how well the model ranks individuals from a subgroup relative to everyone else.

While both the gCI and gAUC and the xCI and xAUC account for cross-group comparisons, the gCI and gAUC offer a more straightforward and interpretable summary. The xCI and xAUC provide a detailed matrix of pairwise discrimination values between all subgroup pairs (eg, 9 values for 3 race groups), capturing how the model ranks individuals across specific group combinations. In contrast, the gCI and gAUC yield a single value per group, summarizing discrimination involving that group across all other subgroups. Notably, the gCI and gAUC for a given group lie within the set of corresponding xCI and xAUC values, representing an aggregated effect of both within-group and cross-group comparisons. This aggregation makes the gCI and gAUC more intuitive and easier to interpret when assessing overall model performance for a specific group.

Overall, these results suggest that the gCI and gAUC are not simply restatements of subgroup-specific CI and AUC. Rather, they reflect a broader and more interpretable notion of fairness in discrimination by summarizing each subgroup’s discrimination performance using a single metric.

## Discussion

### Principal Findings

In this work, we proposed 2 novel and intuitive metrics to assess model discrimination for specific subgroups. Unlike the traditional within-group CI and AUC, which evaluate the model’s ability to rank individuals only within the same subgroup, the gCI and gAUC assess how well the model discriminates for individuals in a target group relative to the overall population. By incorporating both within-group and cross-group comparisons, these metrics provide a more complete picture of subgroup-level performance.

The distinction between within-group and cross-group comparisons is particularly important in real-world applications, where predictive models are deployed across diverse populations and decisions are made in a shared environment. In such settings, cross-group ranking accuracy is essential to ensuring that individuals from different groups receive equitable and reliable predictions. Compared to the traditional within-group CI and AUC, the group-specific metrics gCI and gAUC incorporate cross-group comparisons, providing a more comprehensive assessment of discrimination for each subgroup.

While the xCI and xAUC also account for cross-group ranking, their pairwise nature results in a number of metrics that scale quadratically with the number of groups, complicating interpretation. On the other hand, the gCI and gAUC evaluate performance over all comparable pairs in which at least one individual belongs to group *m* (ie, *G*_*i*_=*m* or *G*_*j*_=*m*), effectively summarizing how well the model ranks individuals from group *m* relative to others. Importantly, this approach treats comparisons symmetrically as it does not distinguish whether the group-*m* individual appears earlier or later in the pair, which simplifies the aggregation process. This omission contributes to the interpretability and tractability of the gCI and gAUC compared to xCI and xAUC.

Beyond their statistical appeal, these metrics can be readily integrated into downstream decision-making processes. For example, gCI and gAUC can be combined with utility-based frameworks to evaluate how predictive performance translates into fair outcomes across groups. Their intuitive definitions and interpretable probabilistic meanings make them well suited for both technical evaluation and policy communication.

This work has several limitations. First, we did not fully investigate the theoretical foundations and statistical properties of gCI and gAUC in the present study. A more comprehensive treatment of these measures, including their statistical consistency, variance estimation, and behavior under data imbalance, is beyond the scope of this paper and will be addressed in future work. Second, fairness is a multifaceted concept that cannot be fully characterized by a single metric. gCI and gAUC assess one specific aspect of fairness by summarizing discrimination performance at the group level, but they do not capture other important fairness dimensions such as calibration. Accordingly, these metrics should not be interpreted as stand-alone or comprehensive measures of fairness, nor should they replace more detailed subgroup-specific error analyses and other complementary fairness evaluations. Importantly, empirical estimates of gCI and gAUC are highly uncertain and should be interpreted with caution when the group in question is small even when the overall sample size across all groups is large. Confidence intervals may be obtained via the method by DeLong et al [17] or bootstrapping.

We also aim to incorporate these metrics into broader fairness-aware modeling frameworks to support equitable model development.

### Conclusions

Our proposed group-level extensions of the CI and AUC, the gCI and gAUC, provide a single statistic that summarizes the fairness of model discrimination performance for a given subgroup while remaining sensitive to cross-group ranking disparities. Applied to the PREVENT equation, they revealed key differences in performance between subgroups not easily apparent from existing metrics. Our results illustrate a practical, interpretable approach for trustworthy evaluation of the discrimination performance of clinical prediction models.

## Supplementary material

10.2196/85822 Multimedia Appendix 1 Mathematical properties of the proposed group-level metrics.

## Abbreviations

ASCVD: atherosclerotic cardiovascular disease
AUC: area under the receiver operating characteristic curve
CI: concordance index
gAUC: group-level area under the receiver operating characteristic curve
gCI: group-level concordance index
PREVENT: Predicting Risk of Cardiovascular Disease Events
xAUC: cross-group area under the receiver operating characteristic curve
xCI: cross-group concordance index
