# Supplementary material for: A Simplified Metric to Streamline Between-Group Fairness Assessment for Predictive Models: Algorithm Development and Evaluation Study
Source: JMIR AI. 2026 Aug 24;5:e85822. doi: 10.2196/85822 (PMC13502933; doi:10.2196/85822)
Supplement: Supplementary file 1 [file ai-v5-e85822-s001.pdf]

Appendix for "A simplified metric to streamline between-group fairness assessment for predictive models"

Haoyuan Wang, MS; Chuan Hong, PhD; Michael Pencina, PhD;
Matthew Engelhard, MD, PhD

1 Properties of Group-Level Metrics

This appendix summarizes several theoretical and interpretive properties of the proposed group-level discrimination metrics, $gCI(m)$ and $gAUC(m)$. Throughout, let R_i denote the model risk score for individual i. For time-to-event outcomes, let o_i be the observed time, $y_i \in \{0,1\}$ the event indicator, and define the standard comparable-pair set and concordant-pair set

$$S^{CI} = \{(i,j) : y_i = 1,\ o_i < o_j\}, \qquad Sc^{CI} = \{(i,j) \in S^{CI} : R_i > R_j\}.$$

For binary outcomes, let $Y_i \in \{0,1\}$ be the label and define

$$S^{AUC} = \{(i,j) : Y_i = 1,\ Y_j = 0\}, \qquad Sc^{AUC} = \{(i,j) \in S^{AUC} : R_i > R_j\}.$$

Let $G_i \in \mathcal{G}$ indicate membership in one of $k = |\mathcal{G}|$ groups. For any $m, n \in \mathcal{G}$, the directional cross-group comparable-pair sets are

$$SxCI(m,n) = \{(i,j) : y_i = 1,\ o_i < o_j,\ G_i = m,\ G_j = n\},$$
$$SxAUC(m,n) = \{(i,j) : Y_i = 1,\ Y_j = 0,\ G_i = m,\ G_j = n\}$$

with concordant subsets $ScxCI(m,n) = \{(i,j) \in SxCI(m,n) : R_i > R_j\}$ and $ScxAUC(m,n) = \{(i,j) \in SxAUC(m,n) : R_i > R_j\}$. The corresponding estimators are $\widehat{xCI}(m,n) = |ScxCI(m,n)|/|SxCI(m,n)|$ and $\widehat{xAUC}(m,n) = |ScxAUC(m,n)|/|SxAUC(m,n)|$. The group-level comparable-pair sets proposed in the main text are

$$SgCI(m) = \{(i,j) : y_i = 1,\ o_i < o_j,\ (G_i = m) \text{ or } (G_j = m)\},$$
$$SgAUC(m) = \{(i,j) : Y_i = 1,\ Y_j = 0,\ (G_i = m) \text{ or } (G_j = m)\},$$

with concordant subsets $ScgCI(m) = \{(i,j) \in SgCI(m) : R_i > R_j\}$ and $ScgAUC(m) = \{(i,j) \in SgAUC(m) : R_i > R_j\}$, yielding $\widehat{gCI}(m) = |ScgCI(m)|/|SgCI(m)|$ and $\widehat{gAUC}(m) = |ScgAUC(m)|/|SgAUC(m)|$.

1.1 Relationship to xCI and $xAUC$

A key property of the group-level estimators is that they aggregate the directional cross-group quantities $\widehat{xCI}$ and $\widehat{xAUC}$ through weights determined by the number of comparable pairs contributed by each group pairing. This follows from the decomposition of the group-level comparable-pair sets:

$$SgCI(m) = SxCI(m,m) \;\cup \bigcup_{n \in \mathcal{G}\setminus\{m\}} \Big(SxCI(m,n) \;\cup\; SxCI(n,m) \Big),$$

and analogously,

$$SgAUC(m) = SxAUC(m,m) \;\cup \bigcup_{n \in \mathcal{G}\setminus\{m\}} \Big(SxAUC(m,n) \;\cup\; SxAUC(n,m) \Big).$$

Define the directional pair counts $N_{ab}^{CI} = |SxCI(a,b)|$ and $C_{ab}^{CI} = |ScxCI(a,b)|$. Then the group-level estimator admits the exact identity

$$\widehat{gCI}(m) = \frac{C_{mm}^{CI} + \sum_{n \neq m} \left(C_{mn}^{CI} + C_{nm}^{CI} \right)}{N_{mm}^{CI} + \sum_{n \neq m} \left(N_{mn}^{CI} + N_{nm}^{CI} \right)}.$$

Writing $\widehat{xCI}(a,b) = C_{ab}^{CI}/N_{ab}^{CI}$ (whenever $N_{ab}^{CI} > 0$), we obtain the weighted-aggregation form

$$\widehat{gCI}(m) = w_{mm}^{CI}\, \widehat{xCI}(m,m) + \sum_{n \neq m} w_{mn}^{CI}\, \widehat{xCI}(m,n) + \sum_{n \neq m} w_{nm}^{CI}\, \widehat{xCI}(n,m),$$

where the weights are determined by the proportions of comparable pairs,

$$w_{mm}^{CI} = \frac{N_{mm}^{CI}}{|SgCI(m)|}, \qquad w_{mn}^{CI} = \frac{N_{mn}^{CI}}{|SgCI(m)|}\ (n \neq m), \qquad w_{nm}^{CI} = \frac{N_{nm}^{CI}}{|SgCI(m)|}\ (n \neq m),$$

and satisfy $w_{mm}^{CI} + \sum_{n \neq m} \left(w_{mn}^{CI} + w_{nm}^{CI} \right) = 1$. An entirely parallel decomposition holds for $\widehat{gAUC}(m)$ by defining $N_{ab}^{AUC} = |SxAUC(a,b)|$, $C_{ab}^{AUC} = |ScxAUC(a,b)|$, and $\widehat{xAUC}(a,b) = C_{ab}^{AUC}/N_{ab}^{AUC}$, which yields

$$\widehat{gAUC}(m) = w_{mm}^{AUC}\, \widehat{xAUC}(m,m) + \sum_{n \neq m} w_{mn}^{AUC}\, \widehat{xAUC}(m,n) + \sum_{n \neq m} w_{nm}^{AUC}\, \widehat{xAUC}(n,m),$$

with weights defined analogously from the corresponding N_{ab}^{AUC} counts.

This representation provides an immediate interpretive consequence: $\widehat{gCI}(m)$ and $\widehat{gAUC}(m)$ are convex combinations of within-group and cross-group components, and therefore they lie within the

range of the contributing directional cells. In particular, whenever the relevant cell estimates are defined, the group-level estimate cannot be smaller than the minimum nor larger than the maximum among $\{\widehat{xCI}(m,m)\} \cup \{\widehat{xCI}(m,n), \widehat{xCI}(n,m) : n \neq m\}$, and analogously for AUC. This clarifies why $gCI(m)$ can be viewed as a single-number summary of the m-th row and column of the xCI matrix, with weights induced by the prevalence of comparable pairs.

1.2 U-statistic interpretation and large-sample behavior

The classical CI and AUC are order-two U-statistics in the sense that they estimate probabilities of correct ranking for randomly drawn comparable pairs. The same structure carries over to the proposed group-conditioned estimators. For fixed m, define the group-level comparability indicator for CI as

$$\Delta_m^{CI}(i,j) = \mathbb{I}(y_i = 1,\ o_i < o_j)\, \mathbb{I}(G_i = m \text{ or } G_j = m),$$

and the corresponding concordance indicator

$$\Gamma_m^{CI}(i,j) = \mathbb{I}(R_i > R_j)\, \Delta_m^{CI}(i,j).$$

Then $|SgCI(m)| = \sum_{i \neq j} \Delta_m^{CI}(i,j)$ and $|ScgCI(m)| = \sum_{i \neq j} \Gamma_m^{CI}(i,j)$, so $\widehat{gCI}(m)$ is the ratio of two order-two pairwise sums, each of which can be expressed as a (possibly asymmetric) U-statistic kernel applied to pairs of observations. The same construction applies to $\widehat{gAUC}(m)$ by replacing the CI comparability event with $\mathbb{I}(Y_i = 1, Y_j = 0)$. Consequently, $\widehat{gCI}(m)$ and $\widehat{gAUC}(m)$ inherit standard large-sample properties of U-statistic–based discrimination estimators: under regularity conditions ensuring stable limits for the relevant pairwise probabilities, the numerator and denominator concentrate around their expectations, and asymptotic normality for the ratio follows from joint asymptotic normality of the pairwise sums together with a delta-method argument.

For practical inference, these results motivate uncertainty quantification strategies that are standard for discrimination metrics defined through comparable pairs. In particular, nonparametric bootstrap resampling at the individual level directly propagates uncertainty from both the concordant and comparable pair counts and yields confidence intervals for $\widehat{gCI}(m)$ and $\widehat{gAUC}(m)$ without requiring closed-form variance derivations. Influence-function or U-statistic variance estimators can also be developed by applying the usual Hájek projection machinery to the pairwise kernels, noting that the group-level restriction enters only through the additional indicator $\mathbb{I}(G_i = m \text{ or } G_j = m)$.

1.3 Sensitivity to group-size imbalance

The weighted-aggregation identity in Appendix 1.1 makes explicit how group-size imbalance influences both the estimand and the precision of group-level metrics. Because the weights w_{ab}^{CI} and w_{ab}^{AUC} are proportional to the numbers of comparable pairs N_{ab}^{CI} and N_{ab}^{AUC}, the contribution of each directional cell depends on how frequently that type of comparison arises in the data. When group m is small, within-group comparable pairs N_{mm}^{CI} (or N_{mm}^{AUC}) can be limited, so $\widehat{gCI}(m)$ and $\widehat{gAUC}(m)$ will be driven primarily by cross-group comparisons with groups that produce many comparable pairs with m. This behavior is aligned with the intended interpretation of group-level discrimination in shared decision environments, where individua
